# Introduction to the Special Issue on Early Evolution and the Last Common Ancestor

**DOI:** 10.1007/s00239-024-10208-6

**Published:** 2024-09-20

**Authors:** Arturo Becerra, Aaron D. Goldman

**Affiliations:** 1 Facultad de Ciencias, UNAM, Mexico, Mexico; 2 Department of Biology, Oberlin College, Oberlin, OH USA; 3 Blue Marble Space Institute of Science, Seattle, WA USA

## Abstract

The early evolution of life spans an extensive period preceding the emergence of the first eukaryotic cell. This epoch, which transpired from 4.5 to 2.5 billion years ago, marked the advent of many fundamental cellular attributes and witnessed the existence of the Last Common Ancestor (LCA) of all life forms. Uncovering and reconstructing this elusive LCA's characteristics and genetic makeup represents a formidable challenge and a pivotal pursuit in early evolution. While most scientific accounts concur that the LCA resembles contemporary prokaryotes, its precise definition, genome composition, metabolic capabilities, and ecological niche remain subjects of contentious debate.

The early evolution of life covers an extensive period between 4.5 and 2.5 billion years ago. It was a time with many fundamental evolutionary events, including prebiotic chemistry, the origin of life, the assembly of elementary metabolic pathways, and the origin of cellular types. This period also marked the origin of many primary cellular attributes and witnessed the existence of the Last Universal Common Ancestor (LUCA) of all extant organisms. Discovering and reconstructing the characteristics and genetics of this ancestor represents a formidable challenge and a fundamental quest in early evolution. While most scientific accounts agree that the LUCA resembles contemporary prokaryotes in its level of cellular complexity, its precise genomic composition, metabolic capabilities, and ecological niche remain topics of contentious debate.

Since Charles Darwin wrote that “every organic being that has ever lived on this Earth may descend from some primordial form,” modern biology has strengthened this idea. Advances in molecular cladistics and comparative genomics have indicated that all living organisms can be included in a primary monophyletic tree. This phylogenetic tree, first derived from analysis of 16S/18S rRNA sequences (Woese et al., 1990), points to a shared origin from a single common ancestor. This ancestral form laid the foundation for the universal genetic code, the central mechanisms of genome replication and gene expression, fundamental anabolic pathways, and membrane-associated ATPase-mediated energy production observed in all domains of life (Becerra et al. 2007). The LUCA, therefore, represents a critical point in the early history of life, a point that we, as modern biologists, are privileged to investigate using comparative methods.

In recent decades, significant efforts have been made to understand the nature of the LUCA. Scientists have worked to estimate the time of the emergence of the LUCA (Betts et al. 2018), reconstruct its genome and proteome (Becerra et al. 2007; Goldman et al. 2013), and predict its metabolism (Weiss et al. 2016; Goldman et al. 2023; Ledford and Meredith 2024), habitat (Weiss et al. 2016) and ecological context (Krupovic et al. 2020). These studies have provided insights into the characteristics of the LUCA and its environment, offering a window into the early stages of life on Earth and helping to resolve the complex processes that gave rise to the diversity of life we see today.

For this reason, we invited several experts to present findings and perspectives from their research in this special issue. As a result, we received nine articles in which readers can find new approaches to reconstructing the characteristics of LUCA, perspectives on the nature of its genome and membrane, the proximity of LUCA to simpler life forms, and the historical development of the LUCA concept. The more than 20 authors who participated in these works included students, early career scientists, and pioneers in the field.

In his article, “Perspective: Protocells and the Path to Minimal Life”, Dave Deamer (2024) describes the initial stages that lead to populations of protocells capable of undergoing selection and evolution. The stages incorporate knowledge of the chemical and physical properties of organic compounds, self-assembly of membranous compartments, and non-enzymatic polymerization of amino acids and nucleotides, followed by encapsulation of polymers to produce protocell populations. His conclusions are based on laboratory simulations of cyclic hydrothermal conditions on the prebiotic Earth. This work provides a perspective on the very early stages of evolution, following the origin of life but prior to the LUCA, to better understand the evolutionary path from non-life to minimal life forms.

Greg Fournier offers another perspective on the evolution of life before and at the time of the LUCA. In his article, “Stem Life: A Framework for Understanding the Prebiotic-Biotic Transition,” Fournier (2024) draws on concepts from paleontology and phylogenetics to portray the complex web of lineages that must have been present throughout the early evolution of life, from its origin to periods following the last universal common ancestor. Paleontological research has repeatedly shown that the last common ancestors of more recent groups of extant organisms lived contemporaneously with other taxa that have no living descendants and that the ecology and evolution of extant groups depended upon these extinct lineages. For the same reason, Fournier argues that the diversity of the earliest prebiotic and biotic systems extended far beyond the ancestors of the LUCA and was essential to its emergence.

This issue also includes historical perspectives of the LUCA concept, analyzing different conceptual frameworks such as the progenote or cenancestor are analyzed. For example, Forterre (2024) draws a connection to the biosphere in which the LUCA and contemporaries lived, which was the product of a long cellular evolution from the origin of life to a proposed second age of the RNA world. He also suggests that the LUCA had a simpler structure than modern organisms, which may have contributed to faster evolution at that time. Forterre claims that the LUCA had an RNA genome and basic metabolic functions and was likely a mesophile or moderate thermophile.

Delaye (2024) analyzed how different methodologies identify sets of ancient proteins that only partially overlap. He discusses how a detailed molecular evolutionary analysis of the reverse gyrase protein family has modified previous inferences about a hyperthermophilic LUCA based mainly on automated bioinformatic pipelines. He concludes by addressing the importance of developing a database for studying genes and proteins traceable to LUCA and previous stages of cellular evolution.

Rivas and Fox (2024) provide another historical review comparing ten major LUCA reconstructions focusing on the translation machinery. They note that despite methodological differences in LUCA reconstructions, they broadly suggest that the LUCA had a nearly complete translation system with a genetic code in place, including critical components such as ribosomal proteins and transcription factors like IF-2, EF-G, EF-Tu, and aminoacyl tRNA synthetases that are consistently identified across LUCA reconstructions. The authors conclude that the LUCA was, therefore, not a progenote, i.e., an organism with a less well-defined linkage between its genotype and phenotype.

In a research article entitled “Volatile Organic Compound Metabolism on Early Earth,” authors Ledford and Meredith (2024) use a previously published LUCA proteome (Crapitto et al. 2022) to estimate the biogenic volatile organic compounds that would have been produced by the metabolism of the LUCA. They identify 27 volatile compounds that were likely produced by the LUCA and compare these compounds to those of modern organisms. These results suggest that, despite some differences between the LUCA volatilome and those of modern organisms, biogenic volatile compound cycling was possibly taking place on early Earth.

In their paper, “Evolution of Cellular Organization Along the First Branches of the Tree of Life,” Kailing et al. (2024) use the same previously published LUCA proteome (Crapitto et al. 2022) alongside reconstructed proteomes of the last archaeal common ancestor (Williams et al. 2017) and the last bacterial common ancestor (Coleman et al. 2021) in order to reconstruct the protein functions associated with biological membranes and track the evolution of those functions from the LUCA to its early descendants. There has been disagreement in the field about when cellularity emerged, some arguing that it was an essential component of the origin of life and others arguing that it did not evolve until after the time of the LUCA. Kailing et al. show that cellularity was well in place by the time of the LUCA but that the fundamental process of phospholipid biosynthesis could not be reconstructed in the LUCA proteome, likely due to the current diversity of phospholipids across the tree of life.

Caetano-Anollés et al. (2024) address the emergence and evolution of loop prototypes, traced through a phylogenomic reconstruction of superkingdoms and viruses. This research identified six distinct evolutionary phases, each marked by the accumulation of strategic prototypes that shaped the structures and functions of common ancestors. Furthermore, they introduce and promote two definitions of universal ancestors: the Last Universal Common Ancestor (LUCA) of cells and viruses and the Last Universal Cellular Ancestor (LUCellA), which were structurally and functionally complex and served as key points in the evolutionary progression of cellular life. Contrary to most conclusions from previous reconstructions (including Rivas and Fox in this issue), they propose that the LUCA lacked ribosomal biosynthetic machinery. In contrast, LUCellA lacked essential DNA biosynthesis and modern transcription. According to their analysis, the first proteins depended on RNA to hold genetic information, transferring LUCA to the early stages of the evolutionary process.

The papers in this special issue's conclusions show that the nature of LUCA is still a subject of intense controversy. While Forterre, Caetano-Anolles, et al., or Deamer suggest a LUCA that looks simpler than modern cells, e.g., possibly resembling stages more closely associated with the origin of life, the other articles reflect the current consensus that the LUCA was similar in its cellular complexity and core molecular functions to a modern bacterium (Lazcano et al. 1992). Cottom-Salas et al. (2024) address this controversy by examining the chemical nature of the genome of the LUCA, i.e., RNA or DNA. Previous authors have argued that AlkB-mediated methylation repair of alkylated RNA molecules may be interpreted as evidence of an ancestral RNA genome. Cottom et al., however, show that the repair of methylated RNA molecules results from the enzyme–substrate ambiguity and does not necessarily indicate that the last common ancestor had an RNA genome.

As this special issue was in its final stages of development, a major study on the last universal common ancestor was published by Moody et al. (2024) in the journal *Nature Ecology and Evolution*. This study provides what is likely to be a high-quality estimate of the LUCA proteome as well as new evidence for the timing and ecology of the LUCA. We include a summary of the study in this special issue entitled “A New View of the Last Universal Common Ancestor” (Goldman and Becerra 2024).

The LUCA is a complex subject to study due to its vast distance from modern life in terms of time and evolutionary history. This special issue offers many new perspectives on the LUCA, its predecessors, and its early descendants. We hope that this collection of articles advances our understanding of this crucial stage in early evolution and, in doing so, provides greater insight into life as we know it today.
